# Hijacking of Endocrine and Metabolic Regulation in Cancer and Diabetes

**DOI:** 10.1155/2015/386203

**Published:** 2015-07-09

**Authors:** Eileen M. McGowan, Ann Simpson, James McManaman, Viroj Boonyaratanakornkit, Anandwardhan A. Hardikar

**Affiliations:** ^1^Medical and Molecular Biosciences, University of Technology Sydney, Sydney, NSW 2007, Australia; ^2^Division of Basic Reproductive Sciences, Graduate Program in Integrative Physiology, University of Colorado School of Medicine, Denver, CO 80045, USA; ^3^Department of Clinical Chemistry, Faculty of Allied Health Sciences, Chulalongkorn University, 154 Rama I Road, Wangmai, Pathumwan, Bangkok 10330, Thailand; ^4^NHMRC Clinical Trials Centre, University of Sydney, NSW 2006, Australia

This issue features articles related to endocrine and metabolic regulation in cancer and diabetes. The modern diet, rich in fat and refined carbohydrates, is creating worldwide obesity and diabetes epidemics. As early as 1959, Joslin and colleagues speculated about an association between diabetes and cancer, and current epidemiologic evidence indicates an increased risk of various types of cancer, with increased morbidity, in diabetes patients. The underlying mechanisms for this association have not been fully elucidated as yet.

Cancer, diabetes, and obesity are all chronic diseases with common discernible alterations in metabolic processes. A sweet tooth and alterations in the glucose metabolism are common underlying themes. As scientists uncover more evidence to support this idea, the current research focuses on preventative strategies and treatments targeting disturbances in common cellular metabolic pathways. Some of the drugs used clinically to treat diabetes have been shown to have antitumour effects, as highlighted in the review of N. Turner et al. (University of New South Wales, Australia).

However, targeted treatments are not so easily implemented and have some inherent complexities, which derive from the possibility that some diabetes treatments may alter cancer risk. Alternatively, some cancer treatments may be incompatible with diabetes therapies. Thiazolidinediones and metformin are two common diabetes treatments which show promise as cancer therapeutics. In this edition, E. Fröhlich and R. Wahl (University of Tuebingen, Germany) discuss thiazolidinediones as treatments for multiple cancer types whilst the review by D. Hatoum and E. M. McGowan (University of Technology Sydney, Australia) summarizes recent advances in the use of metformin in comorbidity of diabetes and breast cancer. Although the* in vitro* evidence is compelling, retrospective evidence for comorbidity therapy is controversial. As another option, contemporary research directed at targeting the sphingolipid rheostat holds great promise for diabetes and cancer therapy. The paper by K. Marzec et al. (Kolling Institute, Sydney, Australia) strongly supports a role for targeting sphingosine kinase 1 (SphK1) in combinational treatments for the hard to manage triple negative breast cancers (TNBC), whereas the corresponding paper by N. K. Haass et al. (University of Queensland/University of Technology Sydney, Australia) focuses on the complexities inherent in the use of sphingosine kinase/sphingosine-1-phosphate modulators in the treatment of comorbidity of cancer and diabetes.

It is becoming clear that obesity and cancer is not a random association. A. J. Hoy et al. (University of Sydney, Australia) emphasize that key pathways of fatty acid metabolism are altered in cancer. Associations between obesity, ovarian steroid hormones, and cancer are common themes in two complementary papers by V. Boonyaratanakornkit and P. Pateetin (Chulalongkorn University, Thailand) and K. H. Joung et al. (Chungnam National School of Medicine, North Korea). These papers describe the strong prevalence of cancer in postmenopausal obese women. V. Boonyaratanakornkit and P. Pateetin raise the importance of developing a better understanding of the molecular mechanisms and signalling pathways associated with alterations in endocrine and metabolic regulation in obese women for improved breast cancer treatment.

IGF-1 and insulin are strongly associated with most diabetes related cancers. Several of the papers in this issue focus on the importance of the insulin-like growth factors (IGFs) as novel targets for cancer therapies. K. Marzec et al. concentrate on IGF binding protein 3 (IGFP-3) as a critical target for TNBCs. The original paper authored by the R. Pietras group (UCLA, USA) showed that elevated IGF-2 was concomitant with stimulated estrogen receptor-*β* (ER*β*) and poor overall survival. Furthermore, the paper by H. S. Atreya et al. (Indian Institute of Science, India) advanced the concept of blocking IGF and IGFBPs in cancer therapies.

The scope of this special issue highlights the growing awareness of the link between altered cellular metabolism and the risk of developing diabetes and cancer. Understanding and targeting altered cellular metabolism in individuals with or without diabetes, so as to reduce the risk of developing cancer, form the basis for future combinational treatments.

